# The Flavonoid Naringenin Alleviates Collagen-Induced Arthritis through Curbing the Migration and Polarization of CD4^+^ T Lymphocyte Driven by Regulating Mitochondrial Fission

**DOI:** 10.3390/ijms24010279

**Published:** 2022-12-23

**Authors:** Yue-Peng Jiang, Jun-Jun Wen, Xiao-Xuan Zhao, Yuan-Cheng Gao, Xiao Ma, Si-Yue Song, Yan Jin, Tie-Juan Shao, Jie Yu, Cheng-Ping Wen

**Affiliations:** 1 College of Pharmaceutical Science, Zhejiang Chinese Medical University, Hangzhou 310053, China; 2 Key Laboratory of Neuropharmacology and Translational Medicine of Zhejiang Province, College of Basic Medical Science, Zhejiang Chinese Medical University, Hangzhou 310053, China; 3 Department of Traditional Chinese Medicine (TCM) Gynecology, Hangzhou Hospital of Traditional Chinese Medicine, Zhejiang Chinese Medical University, Hangzhou 310007, China

**Keywords:** rheumatoid arthritis, collagen-induced arthritis, CD4^+^ T lymphocytes, polarization, migration, mitochondrial fission

## Abstract

Rheumatoid arthritis (RA) is a progressive autoimmune disease. Due to local infiltration and damage to the joints, activated CD4^+^ T cells play a crucial role in the progression of RA. However, the exact regulatory mechanisms are perplexing, which makes the effective management of RA frustrating. This study aimed to investigate the effect of mitochondria fission on the polarization and migration of CD4^+^ T cells as well as the regulatory mechanism of NAR, so as to provide enlightenment on therapeutic targets and novel strategies for the treatment of RA. In this study, a collagen-induced arthritis (CIA) model was established, and rats were randomly given saline or naringenin (NAR, 10 mg/kg, 20 mg/kg, 50 mg/kg, i.p.) once a day, before being euthanized on the 42nd day of primary immunization. The pain-like behavior, articular index scores, account of synovial-infiltrated CD4^+^ T cells, and inflammatory factors were investigated in each group. In vitro, spleen CD4^+^ T lymphocytes were derived from each group. In addition, mitochondrial division inhibitor 1 (Mdivi-1) or NAR was added to the cell medium containing C-X-C motif chemokine ligand 12 (CXCL12) in order to induce CD4^+^ T lymphocytes, respectively. The polarization capacity of CD4^+^ T cells was evaluated through the immunofluorescence intensity of the F-actin and myosin light chain phosphorylated at Ser19 (pMLC S19), and the mitochondrial distribution was determined by co-localization analysis of the translocase of outer mitochondrial membrane 20 (TOM20, the mitochondrial marker) and intercellular adhesion molecule 1 (ICAM1, the uropod marker). The mitochondrial fission was investigated by detecting dynamin-related protein 1 (Drp1) and mitochondrial fission protein 1 (Fis1) using Western blot and immunofluorescence. This study revealed that high-dose NAR (50 mg/kg, i.p.) alleviated pain-like behavior and articular index scores, reduced the serum level of interleukin 6 (IL-6) and tumor necrosis factor α (TNF-α), and accounted for CD4^+^ T lymphocytes that infiltrated into the synovial membrane of the CIA group. Meanwhile, NAR (50 mg/kg, i.p.) suppressed the polarization of spleen CD4^+^ T lymphocytes, reduced the redistribution of mitochondria in the uropod, and inhibited the expression of Drp1 and Fis1 in the CIA model. Furthermore, the in vitro experiments confirmed that NAR reduced mitochondrial fission, which in turn inhibited the CXCL12-induced polarization and migration of CD4^+^ T lymphocytes. Our results demonstrated that the flavonoid NAR was a promising drug for the treatment of RA, which could effectively interfere with mitochondrial fission, thus inhibiting the polarization and migration of CD4^+^ T cells in the synovial membrane.

## 1. Introduction

Rheumatoid arthritis (RA) is a chronic progressive autoimmune disease, manifested by synovial inflammation, joint swelling, and the destruction of bone or cartilage, which affects approximately 1% of the global population [1]. Currently, it is a major public health problem, given its serious negative impact on work capacity and life expectancy [2,3]. Due to the complexity and ambiguity of the pathology, significant attention and effort have been devoted to the pathomechanism of RA, among which CD4^+^ T cells, as known as T helper cells, have gained significant attention on account of their crucial roles in the progression of RA.

CD4^+^ T cells are highly mobile and play an important role in adaptive immunity [4]. They continuously circulate between lymph and blood and enter inflammatory sites, which is essential for the host’s immune defense and inflammatory responses [5,6]. Recently, CD4^+^ T cells have been reported to accumulate in the spleen and lymph nodes of CIA mice [7], and circulating CD4^+^ T lymphocyte subsets were also found to be activated in RA patients [8,9]. Moreover, in response to cytokines and chemokines, activated CD4^+^ T cells accumulate in the synovial lumen in order to induce an aggressive phenotype of synovial fibroblasts and other immunocytes, which leads to increased joint inflammation [2,10,11] and triggers the production of osteoclasts that destroy joint anatomy [12,13]. Therefore, modulating T-cell migration to secondary lymphoid organs and local tissues is expected to provide new insight into the treatment of RA. 

Previous studies have demonstrated that increased human CD4^+^ T cell polarization is accompanied by enhanced migration [14]. Cell migration is an active and physically integrated multi-step process [15,16] in which polarization is an essential prerequisite, characterized by the rearrangement of the actin cytoskeleton to form a leading edge in the direction of migration, and a retracting uropod at the posterior of the cell [17]. However, the molecular mechanisms that regulate CD4^+^ T-cell polarization are not yet known. A growing number of studies have shown that mitochondria are involved in the cell polarization [14]. The mitochondrion is a highly mobile and dynamic organelle, and dynamic changes enable mitochondria to accumulate in subcellular regions with high metabolic activity so as to adapt to cellular functions such as polarization and migration [18,19,20]. Studies have demonstrated that, during T-cell development, the disruption of mitochondrial fission can lead to a reduction in the number of thymocytes and mature T cells, as well as a decrease in the migration of mature circulating T cells to secondary lymphoid organs [21]. However, it has not been elucidated whether modulating the mitochondrial fission may interfere the biological characteristics and functions of CD4^+^ T cells in RA patients. Therefore, illustrating the effects of mitochondrial fission on the polarization and migration capability of CD4^+^ T cells in a CIA rat model and in vitro experiments may provide a new target for immunotherapy of RA.

Naringenin (NAR, 4,5,7-trihydroxyflavone) is one of the most vital and plentiful flavonoids widely found in citrus plants [22,23]. NAR exerts remarkable therapeutic potential in several inflammatory diseases in cellular and animal studies, which are attributed to its strong antioxidant properties [24,25,26]. Additionally, NAR is considered a promising drug for the treatment of RA due to its outstanding anti-rheumatoid activity [27,28]. However, its mechanism of action is still obscure [29]. Furthermore, its effect on CD4^+^ T in RA has not been experimentally confirmed. Thus, our study aimed examine the effect of mitochondria fission on CD4^+^ T-cell polarization and migration, as well as the regulatory mechanism of NAR, and to provide enlightenment on therapeutic targets and novel strategies for the treatment of RA.

## 2. Results

### 2.1. NAR Improved Pain-like Behaviors and Inflammation in CIA Model Rats

**Figure 1:**
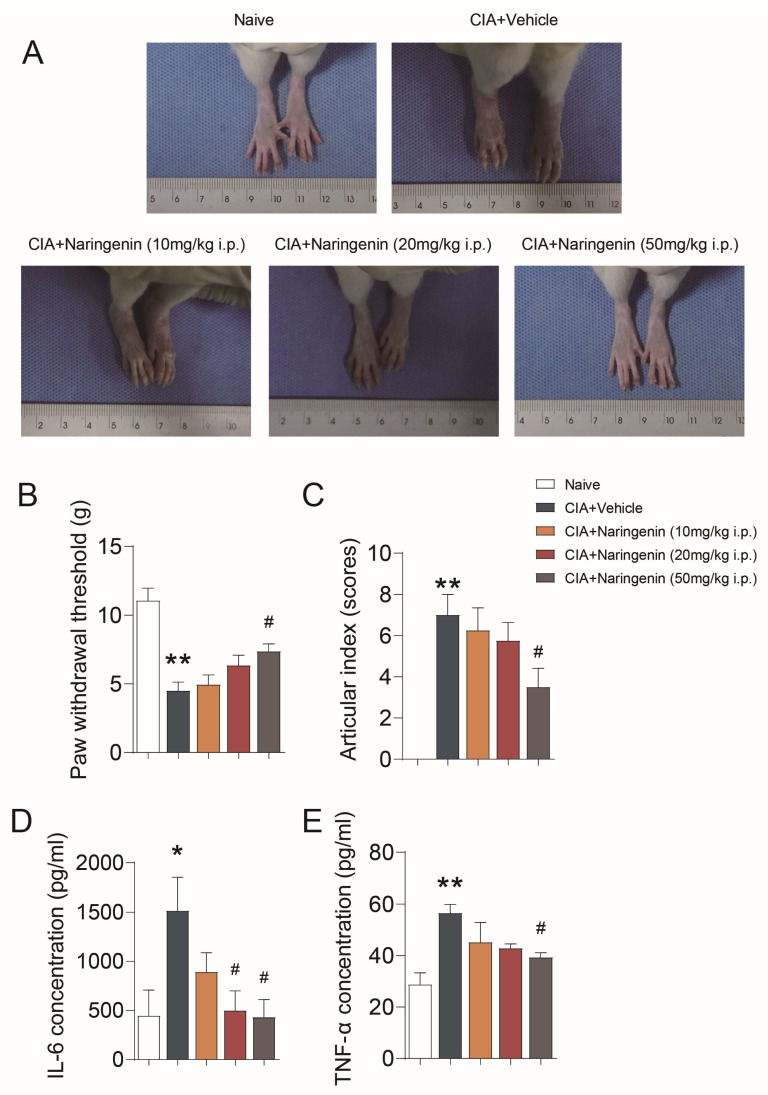
NAR improved pain-like behaviors and inflammation in CIA model rats. (**A**) The redness and swelling of the hind limbs seen in rats in different groups. (**B**) Mechanical pain threshold in CIA rats. (**C**) Arthritic score in CIA rats. ELISA was used to detect IL-6 (**D**) and TNF-α (**E**). * *p* < 0.05 vs. naive group, ** *p* < 0.01 vs. naive group. ^#^ *p* < 0.05 vs. CIA model group, by repeated-measures one-way ANOVA followed by post hoc Dunnett’s multiple comparisons test (n = 6–8 rats per group). In this and subsequent figures, the experimenters were blinded to the treatment condition(s).

CIA rat models were successfully established with high arthritic scores, exhibiting various degrees of redness and swelling in their hind limbs. The NAR intervention on arthritis score and pain-like behavior was dose-dependent. To be specific, high-dose NAR (50 mg/kg) could significantly alleviate the degrees of redness and swelling in the hind limbs (Figure 1A), and could reduce pain thresholds as well as articular index scores (*p* < 0.05) (Figure 1B,C). Moreover, the level of serum interleukin 6 (IL-6) and tumor necrosis factor α (TNF-α) flared up in the CIA model, which was also inhibited by NAR in a dose-dependent manner. More precisely, IL-6 and TNF-α in the high-dose NAR group were significantly lower than those in the CIA group (*p* < 0.05) (Figure 1D,E).

### 2.2. NAR Alleviated the Periarticular Inflammation Scores and Synovial Infiltration of CD4^+^ T Lymphocytes

**Figure 2:**
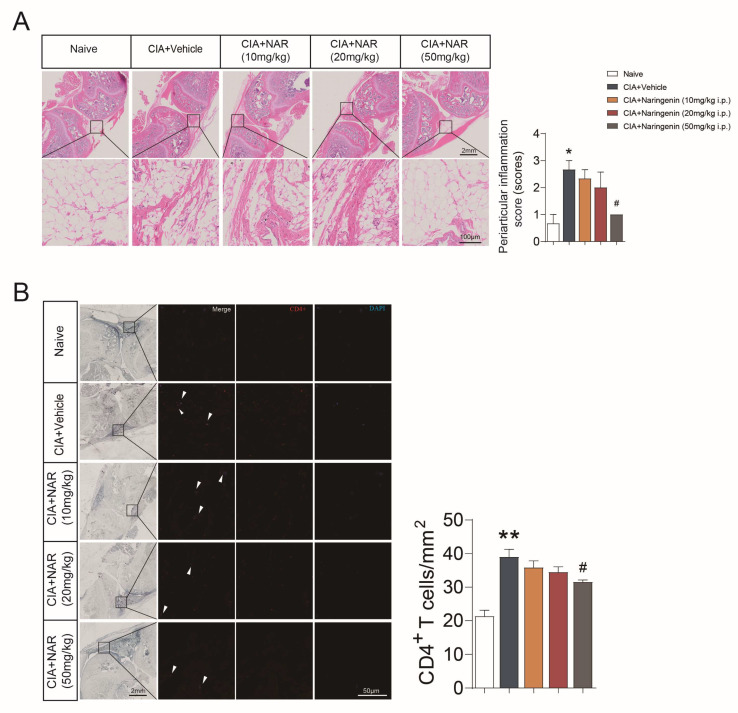
NAR alleviated the periarticular inflammation scores and synovial infiltration of CD4^+^ T lymphocytes. (**A**) The periarticular inflammation scores were calculated by HE staining. (**B**) CD4 as shown by immunofluorescence. * *p* < 0.05, ** *p* < 0.01 vs. naive group, ^#^ *p* < 0.05 vs. CIA model group, using repeated-measures one-way ANOVA followed by post hoc Dunnett’s multiple comparisons test. All scale bars are 2 mm (A upper) and 100 µm (A lower), 2 mm (B left) and 50 µm (B right). The data are presented as means ± SEM (3 sections and 3–5 different fields of each section were randomly selected for semi-quantitative evaluation).

The HE staining results indicated that the periarticular inflammation scores were significantly increased in the CIA group (*p* < 0.05), whereas, NAR (50 mg/kg) significantly mitigated the periarticular inflammation scores (*p* < 0.05) (Figure 2A). The immunofluorescence results showed that the level of synovial CD4^+^ T lymphocytes in the CIA group increased when compared with the naive group (*p* < 0.01), and it was inhibited by NAR in a dose-dependent manner. The NAR (50 mg/kg) group showed alleviated synovial infiltration of CD4^+^ T lymphocytes compared with the CIA group (*p* < 0.05) (Figure 2B).

### 2.3. NAR Ameliorated CD4^+^ T Lymphocyte Polarization in the Spleen of CIA Model Rats

**Figure 3:**
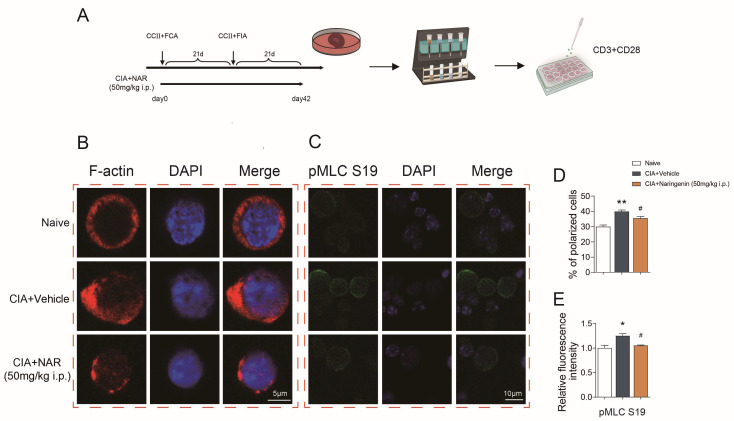
NAR ameliorated CD4^+^ T lymphocyte polarization in the spleen of CIA model rats. (**A**) Flowchart of using magnetic beans to sort CD4^+^ T lymphocytes. (**B**) F-actin shown by immunofluorescence. (**C**) Immunofluorescence showing the expression of pMLC in the CD4^+^ T lymphocyte in different groups. (**D**) The bar chart shows the percentage of polarized cells. (**E**) The bar chart shows the relative fluorescence intensity of pMLC S19. * *p* < 0.05, ** *p* < 0.01 vs. naive group, ^#^ *p* < 0.05 vs. CIA model group, by repeated-measures one-way ANOVA followed by post hoc Dunnett’s multiple comparisons test. All scale bars are 5 µm (**B**) or 10 µm (**C**). The data are presented as means ± SEM (3 sections and 3–5 different fields of each section were randomly selected for semi-quantitative evaluation).

The polar distribution of polymeric F-actin is the first step in the alteration of cell polarization [30]. In order to investigate the polarity pattern of spleen CD4^+^ T cells in CIA mice and the effect of NAR intervention, high-dose NAR (50 mg/kg) was selected for the following experiment according to the above results (Figure 3A). Polymeric F-actin immunofluorescence assay was detected by confocal laser scanning microscopy. The results showed that polymeric F-actin was uniformly expressed over the entire cell surface of spleen CD4^+^ T lymphocytes in the control group while in the CIA group it was asymmetrically distributed, demonstrated by the stronger fluorescence intensity of F-actin in the uropod of the cells. The proportion of cells with an asymmetric distribution of F-actin was significantly increased (*p* < 0.01), suggesting that the quantity of CD4^+^ T cells that underwent polarization was considerably higher in the CIA group. In addition, the results showed that the F-actin distribution in the spleen CD4^+^ T lymphocytes was symmetrical in the high-dose NAR group when compared with the CIA group, and the number of CD4^+^ T cells with asymmetrical red fluorescence distribution was significantly reduced (*p* < 0.05) (Figure 3B,D). Moreover, the expression of myosin light chain phosphorylated at Ser19 (pMLC S19) was significantly decreased in the high-dose NAR group (*p* < 0.05) (Figure 3C,E). All the results suggested that high-dose NAR significantly inhibited spleen CD4^+^ T lymphocyte polarization.

### 2.4. NAR Affected the Mitochondrial Distribution in Spleen CD4^+^ T Cells

**Figure 4:**
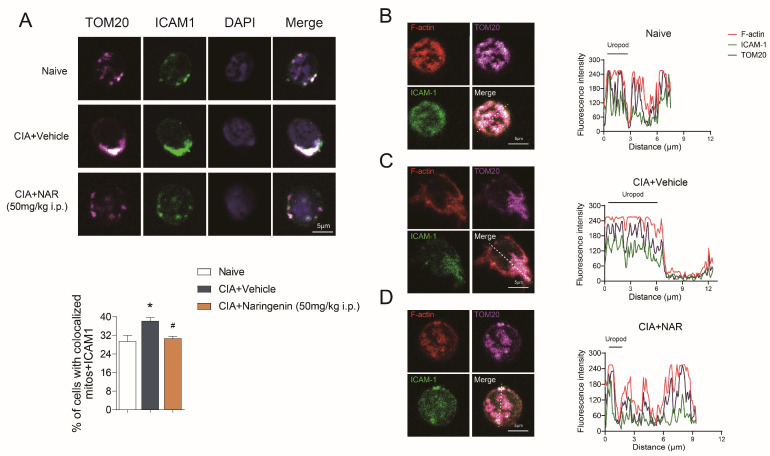
NAR affected the mitochondrial distribution in spleen CD4^+^ T cells. (**A**) Mitochondria (TOM20) and the uropod marker ICAM1 in different groups. The percentage of cells with colocalized mitochondria and ICAM1 is shown in the graph (n = 3 rats per group). Naive group (**B**), CIA + Vehicle group (**C**), CIA+Naringenin (50 mg/kg i.p.) (**D**) CD4^+^ T lymphocytes were stained for the indicated proteins (F-actin (detected using fluorescent phalloidin); ICAM-1; and TOM20) and (left) imaged by confocal microscopy; the uropod is marked with ICAM-1. (Right) Fluorescence intensity analysis was performed from the uropod to the leading edge (L.E.) over the line represented at the merge image. * *p* < 0.05 vs. naive group, ^#^ *p* < 0.05 vs. CIA model group, by repeated-measures one-way ANOVA followed by post hoc Dunnett’s multiple comparisons test. All scale bars are 5 µm. The data are presented as means ±SEM (3 sections and 5 different fields of each section were randomly selected for colocalized analysis).

Intercellular adhesion molecule 1 (ICAM1) staining was used to label the uropods of CD4^+^ T cells and translocase of outer mitochondrial membrane 20 (TOM20) staining, a mitochondrial marker, was used to mark mitochondrial distribution [31]. The mitochondrial distribution in spleen CD4^+^ T cells showed that the ratio of cells with TOM20 and ICAM1 co-localization was significantly higher in the CIA model group when compared with the control group (*p* < 0.05), which suggested that the mitochondria in the CIA model group were located mainly in the uropod of spleen CD4^+^ T lymphocytes. High-dose NAR reduced the ratio of cells with TOM20 and ICAM1 co-localization (*p* < 0.05), as shown in Figure 4A. In addition, we found that in the CIA model group, TOM20 accumulation paralleled that of ICAM1 and F-actin, and they could be detected together in the uropod of migrating CD4^+^ T cells. In contrast, there was low correlation among TOM20, ICAM1, and F-actin in both the control group and the high-dose NAR group, which was characterized by uniform distribution in cells (Figure 4B–D). These results further suggested that NAR could interfere with the polarity and chemotaxis of CD4^+^ T cells.

### 2.5. NAR Improved Mitochondrial Fission of CD4^+^ T Lymphocytes

**Figure 5:**
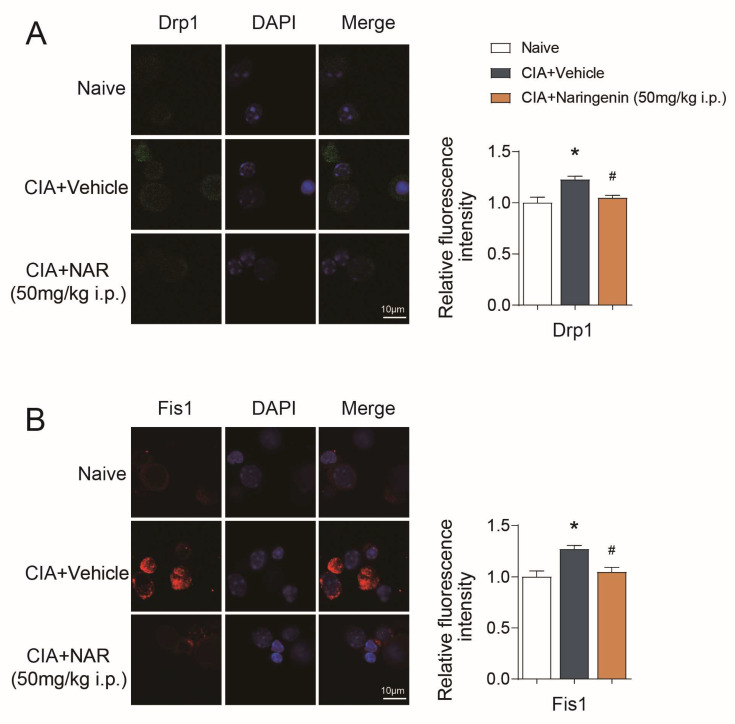
NAR improves mitochondrial fission. (**A**) Immunofluorescence showing the expression of Drp1 in the CD4^+^ T lymphocyte in different groups. (**B**) Immunofluorescence showing the expression of Fis1 in the CD4^+^ T lymphocyte in different groups. * *p* < 0.05 vs. naive group, ^#^ *p* < 0.05 vs. CIA model group, by repeated-measures one-way ANOVA followed by post hoc Dunnett’s multiple comparisons test. All scale bars are 10 µm. The data are presented as means ±SEM (3 sections and 3–5 different fields of each section were randomly selected for semi-quantitative evaluation).

Considering that mitochondrial fission is closely related to cell polarization, we further explored the effects of NAR on mitochondrial fission. Studies have shown that mitochondrial fission protein 1 (Fis1) can promote mitochondrial fission by “promoting the recruitment of dynamin-related protein 1 (Drp1) to mitochondria” [32]. Our results showed that the expression of Drp1 and Fis1 in CD4^+^ T lymphocytes in the CIA model group was increased when compared with the naive group (*p* < 0.05). Moreover, high-dose of NAR alleviates the expression of Drp1 and Fis1 (*p* < 0.05) (Figure 5A,B).

### 2.6. NAR Inhibited the Polarization and Migration of Primary CD4^+^ T Lymphocytes In Vitro by Interfering with Mitochondrial Fission

**Figure 6:**
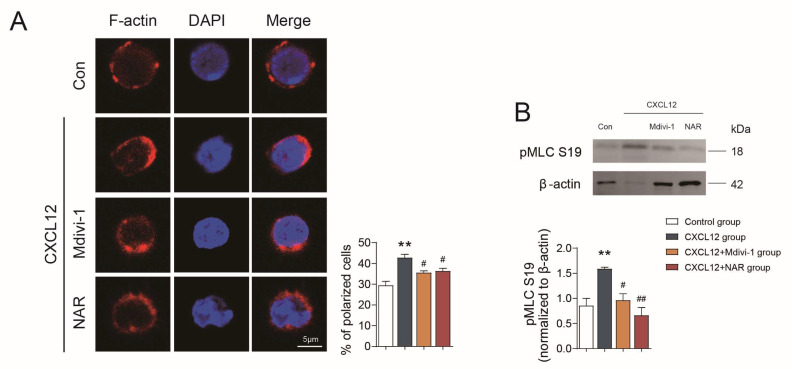
NAR inhibited the polarization of primary CD4^+^ T lymphocytes. (**A**) F-actin of CD4^+^ T lymphocytes measured by immunofluorescence. (**B**) Representative western blots showing the expression of pMLC in the CD4^+^ T lymphocyte in different groups. ** *p* < 0.01 vs. control group, ^#^ *p* < 0.05, ^##^ *p* < 0.01 vs. CXCL12 group, by repeated-measures one-way ANOVA followed by post hoc Dunnett’s multiple comparisons test. All scale bars are 5 µm. The data are presented as means ± SEM (n = 3 mice per group).

**Figure 7:**
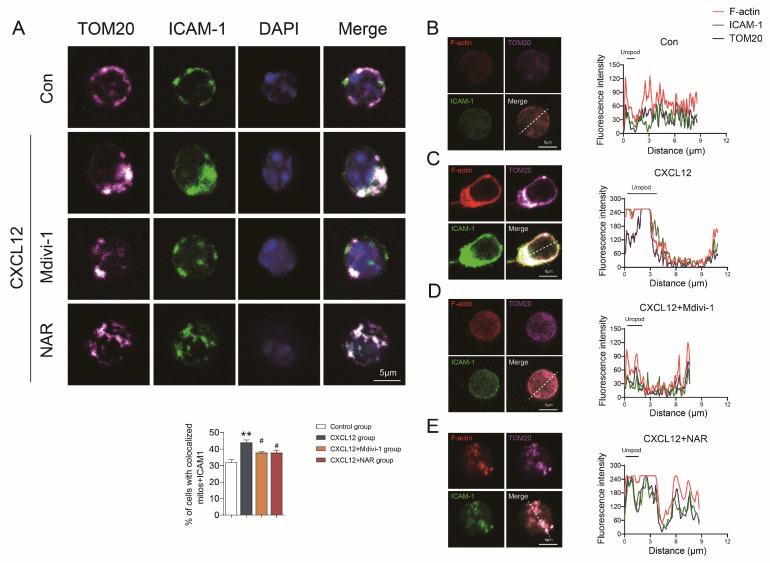
NAR inhibited the mitochondrial distribution and migration of primary CD4^+^ T lymphocytes. (**A**) Mitochondria (TOM20) and the uropod marker ICAM1 in different groups. The percentage of cells with co-localized mitochondria and ICAM1 is reported in the graph (n = 3 mice per group). Control group (**B**), CXCL12 group (**C**), CXCL12 + Mdivi-1 (**D**), CXCL12 + NAR (**E**) CD4^+^ T lymphocytes were stained for the indicated proteins (F-actin (detected using fluorescent phalloidin); ICAM-1; and TOM20) and (left) imaged by confocal microscopy; the uropod is marked with ICAM-1. (Right) Fluorescence intensity analysis was performed from the uropod to the leading edge (L.E.) over the line represented at the merge image. ** *p* < 0.01 vs. control group, ^#^ *p* < 0.05 vs. CXCL12 group, by repeated-measures one-way ANOVA followed by post hoc Dunnett’s multiple comparisons test. All scale bars are 5 µm. The data are presented as means ± SEM (n = 3 mice per group).

To further investigate the performance and mechanism of NAR interfering with the polarization and migration of CD4^+^ T cells in vitro, CD4^+^ T cells were randomly divided into four groups: the control group, the C-X-C motif chemokine ligand 12 (CXCL12) group, the NAR group, and the Mdivi-1 group. First, we identified that CXCL12 significantly induced CD4^+^ T cell polarization and migration, manifested by the increased incidence of CD4^+^ T lymphocytes with polarity distribution of F-actin and the incremental level of pMLC (*p* < 0.01) (Figure 6A,B). The incidence of CD4^+^ T cells with TOM20-ICAM1 co-localization was significantly increased as well (*p* < 0.01) (Figure 7A). In addition, we found that TOM20 accumulation paralleled that of ICAM1 and F-actin, and could they be detected together in the uropod of migrating CD4^+^ T cells in the CXCL12 group (Figure 7B,C). Furthermore, the transwell assay displayed that the migration of CD4^+^ T lymphocytes was significantly increased in the CXCL12 group (*p* < 0.05) (Supplementary Materials Figure S1).

**Figure 8:**
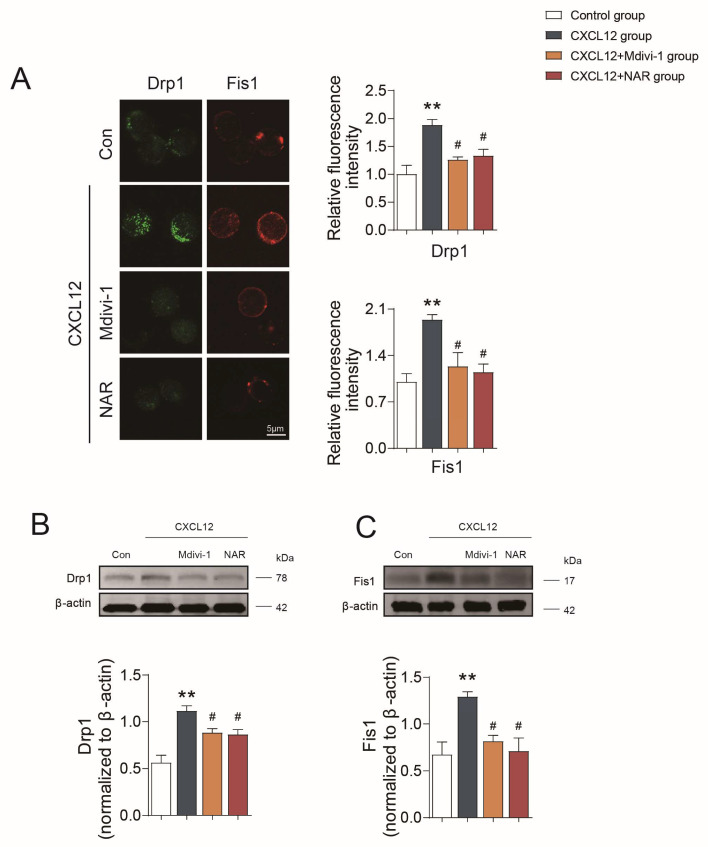
NAR inhibited the mitochondrial fission of primary CD4^+^ T lymphocytes. (**A**) Drp1 and Fis1 as measured by immunofluorescence. Representative Western blots showing the expression of Drp1 (**B**) and Fis1 (**C**) in the CD4^+^ T lymphocyte in different groups. ** *p* < 0.01 vs. control group, ^#^ *p* < 0.05 vs. CXCL12 group, by repeated-measures one-way ANOVA followed by post hoc Dunnett’s multiple comparisons test. All scale bars are 5 µm. The data are presented as means ± SEM (n = 3–4 mice per group, 3 sections and 3–5 different fields of each section were randomly selected for semi-quantitative evaluation).

**Figure 9:**
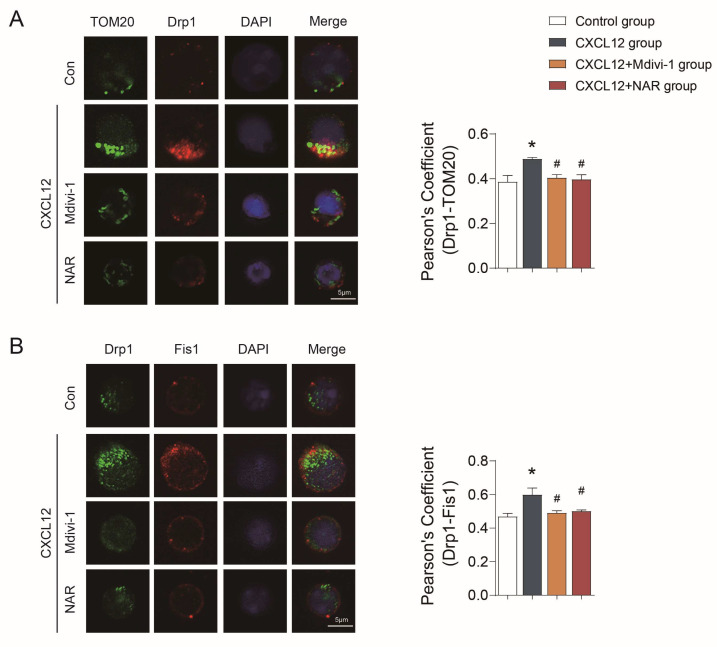
NAR reduced the co-localization of Drp1 with TOM20 as well as Drp1 with Fis1 in primary CD4^+^ T lymphocytes. (**A**) Co-localization of Drp1 with TOM20. (**B**) Co-localization of Drp1 with Fis1. * *p* < 0.05 vs. control group, ^#^ *p* < 0.05 vs. CXCL12 group, by repeated-measures one-way ANOVA followed by post hoc Dunnett’s multiple comparisons test. All scale bars are 5 µm. The data are presented as means ±SEM (n = 3 mice per group).

Subsequently, we further investigated the relationship between mitochondrial fission and the polarization and migration capacity of CD4^+^ T cells. The mitochondrial fission is regulated by Drp1 through its interactions with mitochondrial adaptors including Fis1 [33]. The results confirmed that the levels of Drp1 and Fis1 were significantly increased in the CXCL12 group (*p* < 0.01) (Figure 8A–C), and the co-localization of Drp1 with TOM20, as well as Drp1 with Fis1, was significantly increased (*p* < 0.05) (Figure 9A,B), indicating that the induction of cell polarization and migration by CXCL12 depends on its activation of mitochondrial fission.

In contrast, the results also confirmed that Mdivi-1 could inhibit the expression of Drp1 and Fis1, and reduce the co-localization of Drp1 with TOM20, as well as Drp1 with Fis1 in CD4^+^ T lymphocytes (*p* < 0.05) (Figure 8 and Figure 9). Besides, the polarized CD4^+^ T lymphocytes with polarity distribution of F-actin and high level of pMLC were significantly reduced in the Mdivi-1 treatment group (*p* < 0.05) (Figure 6A,B), and the incidence of cells with TOM20 and ICAM1 co-localization was also decreased (*p* < 0.05) (Figure 7A). In addition, the transwell assay showed that the migration of CD4^+^ T lymphocytes was significantly reduced in the Mdivi-1 treatment group (*p* < 0.05) (Figure S1). Similarly, we found that TOM20 had a low correlation with ICAM1 and F-actin in the Mdivi-1 treatment group and was uniformly distributed in cells (Figure 7D). The above results indicated that the inhibition of mitochondrial fission might contribute to the inhibition of polarization and migration capacity in CD4^+^ T lymphocytes. 

Moreover, we further clarified the effects of NAR on mitochondria fission along with the polarization and migration capacities of CD4^+^ T lymphocytes. The results showed the expression of Drp1 and Fis1 was significantly reduced in the NAR group when compared with the CXCL12 group and the co-localization of Drp1 with TOM20, as well as Drp1 with Fis1, in CD4^+^ T lymphocytes was reduced (*p* < 0.05) (Figure 8 and Figure 9). In addition, the number of polarized CD4^+^ T (*p* < 0.05) and the expression of pMLC (*p* < 0.01) were decreased by NAR (Figure 6A,B). The incidence of cells with TOM20 and ICAM1 co-localization was significantly decreased (*p* < 0.05) (Figure 7A). Moreover, the transwell assay verified that the migration of CD4^+^ T lymphocytes was significantly reduced in the NAR group (*p* < 0.05) (Figure S1). We also found that TOM20 had a low correlation with ICAM1 and F-actin in the NAR group, and was uniformly distributed in cells (Figure 7E). Taken together, the above results indicated that NAR inhibited the polarization and migration of CD4^+^ T lymphocytes by inhibiting mitochondrial fission.

## 3. Discussion

**Figure 10:**
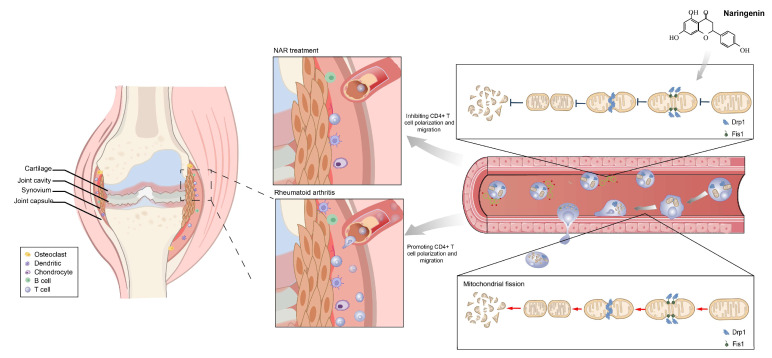
The mechanism of Naringenin alleviates CIA by curbing the migration and polarization of CD4^+^ T lymphocytes driven by regulating mitochondrial fission.

In this study, we demonstrated that NAR was highly effective in improving pain-like behaviors and reducing articular index scores and inflammation factors in CIA rat models. Furthermore, we also uncovered that NAR was capable of inhibiting the polarization state of spleen CD4^+^ T lymphocytes and subsequent synovial infiltration in the CIA model. Our results verified that the inhibition of NAR on CD4^+^ T polarization and migration was closely related to its suppression of mitochondrial fission, thus exerting its anti-inflammatory effects in RA (Figure 10).

CD4^+^ T cell infiltration at the inflammation site is a main characteristic feature of several autoimmune syndromes [34] including RA [35,36]. Accumulated evidence so far has suggested that CD4^+^ T cells rich in local joints of RA patients play a pivotal pathogenic role [37,38]. Xiong et al. further confirmed that lymphocytes infiltrating in patients with RA were mainly CD4^+^ lymphocytes, and most of them were distributed in rheumatoid joints [39]. This shows that CD4^+^ T cells enriched in local joints exert an important effect on the pathomechanism of RA. In this study, we identified that the quantity of CD4^+^ T lymphocytes infiltrating the joints of the CIA model was increased, accompanied by excessive inflammatory factors IL-6 and TNF-α, which was consistent with the work of Dejaco et al. [40]. In addition, we also verified that NAR significantly decreased the quantity of joint-infiltrating CD4^+^ T lymphocytes, and reduced the levels of inflammatory cytokines, which confirmed the exact anti-inflammatory effect of NAR in RA.

Effector CD4^+^ T cells localized in target tissues exert inflammatory effects in their migration to secondary lymphoid-like organs and then trafficking into the locally inflamed tissues. CD4^+^ T cells that migrate to secondary lymphoid-like organs can search for matching antigens displayed by antigen-presenting cells (APCs), and sequentially interrogate the APCs in search of cognate antigens capable of eliciting T cell activation [41,42]. Following activation, CD4^+^ T cells can differentiate into a heterogeneous population of effector T cells that facilitate the generation of specialized immune responses in target tissues [43,44]. It is acknowledged that cell polarization initiates migration [45,46]. Prior to migration, leukocytes undergo polarization with lamellipodia at the leading edge and a uropod at the trailing edge, which enables them to translate cytoskeletal forces into net cell-body displacement [45]. The formation of the uropod is driven by cytoskeleton reassembly, which involves 70 kinds of proteins, such as F-actin, myosin, etc. [47,48]. Fluorescence electron microscopy revealed that the uropod contained a dense network of F-actin microfilaments and a few microtubules [49]. Therefore, the polar distribution of F-actin represents the polarization state of the cells. Here, we stained F-actin specifically by using phalloidin, and the results showed that polarized CD4^+^ T lymphocytes, with a polar distribution of F-actin, were significantly increased in the CIA group. We also detected pMLC, which plays a pivotal role in regulating the ATPase activity of myosin and cytoskeletal architecture, and the results revealed that the expression profiles of pMLC in spleen CD4^+^ T lymphocytes of the CIA group were significantly increased, while high-dose NAR significantly reduced the level of pMLC in CD4^+^ T lymphocytes from the CIA model group. Moreover, NAR significantly interfered with CD4^+^ T lymphocyte polarization in the CIA model group. We further demonstrated that NAR can significantly attenuate the stimulation of CD4^+^ T cell migration by CXCL12 in vitro. Taken together, we inferred that preventing the influx of T cells into inflammatory tissues by NAR was closely bound up with its inhibition of CD4^+^ T cell polarization and migration, thus effectively curbing pathological inflammatory responses, although the specific targets it depends on and the regulatory mechanism of CD4^+^ T cell polarization were still unclear.

Mitochondrial dynamics are important regulators of T cell physiology [50] such as T cell memory development [51] and activation [52], and gather at the uropod of T cells when more energy is required for cell migration [21]. A family of “mitochondria-shaping” proteins regulate the continuous morphology and distribution changes of mitochondria through both fission and fusion events, including profusion proteins such as the large dynamin-like GTPase Opa1 and mitofusin (Mfn) 1 and 2, as well as the pro-fission members such as the cytosolic GTPase Drp1 and its mitochondrial outer membrane protein Fis1. Studies have confirmed that ablation of Drp1 from T cells disrupts their normal extravasation from blood towards secondary lymphoid organs, and also towards an “inflammation site” [53]. Our results revealed that the polarized CD4^+^ T cells in the CIA model group were accompanied by an increase in Drp1 and Fis1 that promotes mitochondrial fission by “facilitating the recruitment of Drp1 to mitochondria” [32,54]. Similarly, as induced by CXCL12 in vitro, the expression of Drp1 and Fis1 was significantly increased. In contrast, the mitochondrial division inhibitor Mdivi-1 reduced Drp1 and Fis1 expression, as well as CD4^+^ T lymphocyte polarization and migration properties. It indicated that mitochondrial fission was closely involved in the polarization and migration of CD4^+^ T cells. Targeting the mitochondrial fission of CD4^+^ T cells is expected to be a new direction for immunosuppressant development in the treatment of RA.

Currently, several drugs are widely used in the treatment of RA, such as nonsteroidal anti-inflammatory drugs, glucocorticoids, and biological drugs. However, various unavoidable side effects diminish the possibility of long-term benefits from the above therapies for RA patients, such as a high risk of infection and cardiovascular disease [55,56]. Thus, the exploration of new strategies has become a common endeavor today. Recently, a network-based pharmacology study predicts NAR as a natural immunomodulator in autoimmune disease by targeting T cells [57]. Its therapeutic role in RA has been corroborated in in vitro and in vivo experiments. Studies have confirmed the administration of NAR is conducive to ameliorating the thickness of the hind paws and the arthritis index scores in experimentally-induced RA in Wistar rats, and diminishing inflammation factors in RAW 264.7 inflammation model rats [27,28]. Similarly, in the present study, we also showed that high-dose NAR improved pain-like behavior and articular index scores and reduced serum IL-6 and TNF-α levels in CIA model rats. Unfortunately, the anti-inflammatory mechanisms of NAR have not been explored comprehensively in published studies so far. Given our confirmation that CD4^+^ T-cell polarization and migration mediated by mitochondria dynamics may be a central mechanism in RA, whether NAR can act on the mitochondrial dynamics of CD4^+^ T cells and thus affect their physiological property in RA remains uncertain. It was reported that NAR blocked the migration of pathogenic T cells into the central nervous system (CNS) in experimental autoimmune encephalomyelitis models [58]. In this study, we demonstrated that NAR reduced the polarization of CD4^+^ T lymphocytes and their infiltration in synovial tissue in CIA model animals. Furthermore, we confirmed that the intervention of NAR on CD4^+^ T lymphocytes correlated with its inhibition of mitochondrial fission. We uncovered that NAR could effectively reduce the expression profile of Drp1 and Fis1, which was supposed to be an essential mechanism for its anti-inflammatory effect in RA.

## 4. Materials and Methods

### 4.1. Reagents and Antibodies

Immunization Grade Bovine Type II Collagen (Cat. No. 20021; Chondrex, Woodinville, WA, USA), NAR (Cat. No. B21596; Shanghai Yuanye Bio-Technology Co., Ltd., Shanghai, China), mitochondrial division inhibitor 1 (Mdivi-1) (Cat. No. SC8028; Beyotime, Nantong, China), recombinant mouse CXCL12/SDF-1α protein (Cat. No. 460-SD-050; R&D Systems, Minneapolis, MN, USA), ultra-LEAF™ purified anti-mouse CD3 antibody (Cat. No. 100238; Biolegend, San Diego, CA, USA), ultra-LEAF™ purified anti-mouse CD28 antibody (Cat. No. 102116; Biolegend), rat-IL-6-HS ELISA kit (Cat. No. EK306HS-96; MultiSciences, Hangzhou, China), rat-TNF-α-HS (Cat. No. EK382HS-96; MultiSciences), mitochondrial membrane potential assay kit with JC-1 (Cat. No. C2006, Beyotime), mitoSOX red mitochondrial superoxide indicator (Cat. No. LX3608; Warbio, Nanjing, China), coraLite^®^594-Phalloidine (red) (Cat. No. PF00003; proteintech, Singapore), MACS buffer (Cat. No. 130-091-221, Miltenyi, Bergisch Gladbach, Germany), CD4^+^ T Cell Isolation Kit (mouse) (Cat. No. 130-104-454, Miltenyi), Transwell (5.0 μm) (Cat. No. 3421, Corning, Corning, NY, USA), poly-lysine coated slides (Cat. No. 188158; LIUSHENG, Nantong, China). Western blot experiments were performed using the following antibodies: rabbit anti-Drp1 monoclonal antibody (1:1000; Cat. No. 8570; CST), mouse anti-Fis1 monoclonal antibody (1:1000; Cat. No. sc-376447; SANTA), rabbit anti-pMLC (Ser-19) monoclonal antibody (1:1000; Cat. No. 3671; CST), rabbit anti-β-actin monoclonal antibody (1:1000; Cat. No. 4970; CST), and goat anti-rabbit IgG (H&L) (1:5000; Cat. No. 926-32211; LI-COR, Lincoln, NE, USA). Immunofluorescence experiments were performed using the following antibodies: rabbit anti-TOM20 antibody (1:200; Cat. No. 42406; CST), mouse anti-ICAM1 antibody (1:200; Cat. No. Ab171123; Abcam, Cambridge, UK), goat anti-rabbit IgG H&L (Alexa Fluor^®^ 488) (1:200; Cat. No. ab150077; Abcam), goat anti-rabbit IgG H&L (Cy3^®^) (1:200; Cat. No. ab6939; Abcam), goat anti-rabbit IgG H&L (Alexa Fluor^®^ 647) (1:200; Cat. No. Ab150083; Abcam), goat anti-mouse IgG H&L (Alexa Fluor^®^ 488) (1:200; Cat. No. ab150113; Abcam), and goat anti-mouse IgG H&L (Alexa Fluor^®^ 647) (1:200; Cat. No. ab150115; Abcam).

### 4.2. Model Preparation and Animal Grouping

Experimental animals: SPF female Wistar rats and male DBA/1 mice were provided by Shanghai SLAC Animal Co., Ltd., Shanghai, China. The production license number of the experimental animal was SCXK (Zhejiang, China) 2017-0005. The animals were raised in the Laboratory Animal Research Center (LARC) of Zhejiang Chinese Medical University, and the license number for the laboratory animals is SYXK (Zhejiang) 2021-0012. At 6–8 weeks of age, the body weight of rats and mice was 180 ± 15 g and 20 ± 2 g, respectively. The ambient temperature fluctuated within 22 ± 1 °C, and the humidity fluctuated between 50% and 60%. Light was 12 h day/12 h night. All animal experiments were conducted by the LARC Animal Ethics Code (Ethics number: IACUC-20220913-12).

- Establishment of a rat model of collagen-induced arthritis (CIA): The rats were fed adaptively until 8 weeks of age, and the model of collagen-induced arthritis was started. A solution of Bovine Type II Collagen-glacial acetic acid at a concentration of 4 mg/mL was prepared in advance and put in the refrigerator at 4 °C under light. The next day, the emulsion (2 mg/mL) was obtained by 1:1 mixing the solution of Bovine Type II Collagen-glacial acetic acid with FIA on ice using a high-speed agitator. The primary immunization was performed on day 0 by subcutaneous injection (200 μL/rat) 3 cm from the tail root of the rats. The second immunization was performed on day 7 by subcutaneous injection (100 μL/rat) 2 cm from the tail root of the rats.
- Establishment of a mouse model of the CIA: The preparation process of Bovine-Type II collagen-glacial acetic acid solution was the same as that of rats. The next day, a high-speed agitator was used to emulsify the Bovine-Type II collagen-glacial acetic acid solution with Freund’s complete adjuvant (FCA) (4 mg/mL) and Freund’s incomplete adjuvant (FIA) at a ratio of 1:1 (FCA was used for the primary immunization, the secondary immunization used FIA) to obtain emulsion, and the final concentration of emulsion was 2 mg/mL. On day 0, the emulsion was injected into the tail of mice for primary immunization (100 μL/mouse), and on day 21, the emulsion was injected into different parts of the back of mice for secondary immunization (100 μL/mouse).
- Rats were divided into five groups: A naive group, a CIA+vehicle group, and CIA+NAR low (10 mg/kg), medium (20 mg/kg), and high dose (50 mg/kg) groups; eight rats per group. Rats were injected intraperitoneally with 40 mg/kg pentobarbital sodium to induce anesthesia and were sacrificed by CO_2_ (100% concentration, for 12 min) on day 42, and the peripheral blood and knee joints were harvested.
- Mice were divided into three groups: A naive group, a CIA + vehicle group and a CIA + NAR (50 mg/kg); 10 mice per group. The mice were sacrificed by CO_2_ (100% concentration, for 3 min) on day 42, and the spleen was harvested.
- Preparation and administration of NAR: According to Zhou’s method [59], NAR was dissolved in solvent (10% DMSO, 10% tween-80, 80% normal saline), and the doses of 10 mg/kg, 20 mg/kg, and 50 mg/kg, respectively, prepared for intraperitoneal injection before clinical use.

### 4.3. Mechanical Pain Threshold

The mechanical pain threshold was measured every seven days from the initial immunization, and the rats were acclimated for 15 min before the test until exploratory behavior was significantly reduced. The test was limited to the mid-plantar portion of the hindlimb. Von Frey hairs (0.4 g, 0.6 g, 1.0 g, 2.0 g, 4.0 g, 6.0 g, 8.0 g, and 15.0 g) in the strength range of 0.4 g to 15.0 g were tested in rats. Normal rats were tested starting at 2.0 g with an interval of more than 5 min. If there was noticeable retraction, flicking, or violent throbbing of the animal’s foot, the von Frey hairs force level was lowered by one step. If no pain behavior was observed, the von Frey hairs power level was increased by one step. The pain threshold was calculated by referring to the modified up-and-down method [60].

### 4.4. Articular Index Scores

The severity of joint inflammation in CIA rats was evaluated back-to-back by three experimental researchers according to the general standard–grade 5 scoring method [61]. Severity is recorded on a scale of 0–4: 0 = no redness or swelling; 1 = slight ankle swelling or redness of the foot; 2 = Progressive swelling, inflammation, and redness from the ankle to the middle of the foot; 3 = swelling and inflammation of the entire foot; 4 = swelling and inflammation of the entire foot and loss of mobility. The maximum rating is 16 points per rat (4 points per limb), and the average value was calculated as the joint score. 

### 4.5. Enzyme-Linked Immunosorbent Assay (ELISA)

Serum samples were collected from the whole blood of rats without anticoagulant, centrifuged at 3000 RPM for 15 min at 4 °C, and the supernatant was taken for detecting serum IL-6 and TNF-α by ELISA according to the corresponding instructions. The working solution, standard, biotin antigen working solution, and horseradish peroxidase-labeled avidin working solution were prepared according to the instructions. Reagents were added to blank, standard, zero, and sample wells. Next, incubation, washing, color development, and termination of the reaction were carried out. Finally, the optical density was measured at 450 nm wavelength by zeroing with a blank hole.

### 4.6. Histologic Assessment of Arthritis

Knee specimens were decalcified and then stained using hematoxylin-eosin (HE). The periarticular inflammation score was made according to the images taken under a high-magnification view of the microscope [62,63]. The scoring criteria for the periarticular inflammation score (mononuclear cell infiltration) was: no inflammatory cell infiltration was defined as 0; <20 was defined as 1; 20–50 was defined as 2; and >50 was defined as 3.

### 4.7. Assessment of Joint Infiltration by CD4^+^ T Lymphocytes

The immunofluorescence method was used to detect the content of CD4^+^ in synovial tissues. Paraffin sections of the synovial tissue of the knee joint of rats were taken, washed, and blocked, and then CD4 antibody (1:200) was added and incubated overnight at 4 °C. Next, the samples were incubated with goat anti-rabbit IgG H&L (Alexa Fluor^®^ 647) in the dark (1:200) for 2 h. After that, the antifade mounting medium with DAPI was added. The CD4^+^ cells were located with a digital pathological section (fluorescence) scanning analyzer (VS120-S6-W, OLYMPUS, Tokyo, Japan) and were analyzed using Image J Software (version 1.53v). Three sections and five different fields of each section were randomly selected for semi-quantitative evaluation as below.

### 4.8. Magnetic Beads Sorting CD4^+^ T Lymphocytes

First, the spleens of mice were obtained from the control group, the CIA group, and the high-dose group (NAR, 50 mg/kg) after euthanasia. Then the tissue was ground and treated with RPMI 1640. After centrifugation, erythrocyte lysate was added into the cells. Then, the mixed cells were obtained by filtration through a 70 μm strainer. Subsequently, MACS buffer and a biotin-antibody cocktail were added for 5 min, and then MACS buffer and anti-biotin microbeads were added for 10 min at 4 °C to isolate CD4^+^ T lymphocytes by LS Columns. Twenty-four-well plates were coated with anti-CD3 (10 μg/mL) (Cat. No. 100238; Biolegend) in sterile PBS, covered and wrapped in parafilm to prevent evaporation and contamination, and then incubated at 4 °C the day before CD4^+^ T lymphocytes were isolated. CD4^+^ T lymphocytes selected from the spleen were cultured in RPMI 1640 complemented with 10% fetal bovine serum and anti-CD28 (2 μg/mL) (Cat. No. 102116; Biolegend) [64].

### 4.9. Evaluation of Spleen CD4^+^ T Lymphocyte Polarization

Cytoskeletal protein reassembly drives cell polarization and F-actin and myosin are representative components of microfilament proteins [65]. Here we stained F-actin with phalloidin (Cat. No. PF00003; Proteintech) and detected the phosphorylation level of myosin light chain (pMLC) to determine the polarization status of CD4^+^ T cells in each group. First, CD4^+^ T cells were coated on polylysine-coated slides (Cat. No. 188158; LIUSHENG), and then the cells were fixed with 4% paraformaldehyde and blocked with BSA (BSA 5 g, 30%Triton X-100 0.5 mL, sodium azide 0.05 g, 1 × PBS) for 2 h. CD4^+^ T lymphocytes were incubated with rabbit anti-pMLC (1:200) overnight at 4 °C, followed by extensive washing and incubated with goat anti-rabbit IgG H & L (Alexa Fluor^®^ 488) for 2 h, and then the cells were incubated with phalloidin for 20 min. After washing, an antifade mounting medium with DAPI was added to seal the images. After that, a confocal laser scanning microscope (LSM880, Zeiss, Jena, Germany) was used to observe and photograph the image. Image J Software was used for polarization percentage analysis and semi-quantitative evaluation of pMLC. 

### 4.10. Mitochondrial Distribution and Fission of Spleen CD4^+^ T Lymphocytes

Immunofluorescence co-localization analysis of TOM20 (the mitochondrial marker) and ICAM1 (the uropod marker) was performed to determine the mitochondria distribution in CD4^+^ T lymphocytes. Mitochondrial fission was detected by immunofluorescence. CD4^+^ T lymphocytes were incubated with primary antibodies: rabbit anti-TOM20, mouse anti-ICAM1 (1:200), rabbit anti-Drp1, and mouse anti-Fis1 (1:200) overnight at 4 °C, followed by extensive washing, then incubated with secondary antibodies: Goat anti-rabbit IgG H & L (Cy3), goat anti-mouse IgG H & L (Alexa Fluor^®^ 488) (1:200), goat anti-rabbit IgG H & L (Alexa Fluor^®^ 488), and goat anti-mouse IgG H & L (Alexa Fluor^®^ 647) (1:200) for 2 h. A confocal laser scanning microscope (LSM880, Zeiss) was used to observe and photograph the images. Image J Software was used to observe the distribution of TOM20 and calculate the co-localization of TOM20 and ICAM1. Semi-quantitative evaluation of Fis1, Drp1, and Pearson correlation coefficient of Drp1-TOM20 and Drp1-Fis1 was performed using Image J Software.

### 4.11. In Vitro Induction of Primary CD4^+^ T Lymphocytes

- Cell polarization induction: Twelve mice in the control group were selected. CD4^+^ T lymphocytes were isolated using MACS cell separation beads and plated at a density of 1.5 × 10^5^ cells/well at 37 °C, 5% CO_2_ in 24-well plates. Finally, cells were incubated with CXCL12 (100 ng/mL) for 6 h at 37 °C [66].
- Cell grouping: CD4^+^ T lymphocytes were divided into the following four groups: CD4^+^ T lymphocyte, CD4^+^ T lymphocyte+CXCL12, CD4^+^ T lymphocyte + CXCL12 + Mdivi-1, and CD4^+^ T lymphocyte + CXCL12 + NAR. NAR treatment was applied overnight at 100 µM [59], while Mdivi-1 treatment was applied overnight at 50 µM, using 0.1% DMSO as vehicle control [67].

### 4.12. Cell Migration Assay

A CD4^+^ T cell migration assay was performed in a Transwell chamber (5.0 μm pore size). The upper chamber was composed of 5.0 × 10^5^ CD4^+^ T lymphocytes from different groups after corresponding intervention and 150 μL RPMI 1640 medium. The lower chamber contained CXCL12 (30 ng/mL) and 600 μL RPMI 1640 medium (containing 10% FBS). The cells were cultured for 24 h at 37 °C in a 5%CO_2_ cell incubator. Next, the upper chamber was discarded and cells in the chamber were observed and counted by microscopes at 100 magnification and Image J Software. We randomly selected 3–5 sections and 5 different fields of each section for semi-quantitative evaluation.

### 4.13. Detection of Protein Expression by Western Blot

CD4^+^ T lymphocytes were isolated by homogenization and sonication in RIPA buffer (Cat. No. P0013B, Beyotime Biotechnology), protease inhibitors (Cat. No. B14002; Bimake, Houston, TX, USA), phosphatase inhibitors (Cat. No. B15002, Bimake), and pierce^TM^ universal nuclease for cell lysis (Cat. No. 88700, Thermo Scientific, Waltham, MA, USA). Proteins were separated by sodium dodecyl sulfate-polyacrylamide gel (12.5%) electrophoresis and then were transferred to nitrocellulose membranes by wet transfer at 300 mA, 80 min. Membranes were incubated in blocking solution (PBS, 0.1% Tween 20, 5% nonfat dried milk, 1 h), then incubated (overnight, 4 °C) with specific antibodies: Rabbit anti-Drp1 monoclonal antibody (1:1000; Cat. No. 8570; CST), mouse anti-Fis1 monoclonal antibody (1:1000; Cat. No. sc-376447; SANTA), rabbit anti-pMLC (Ser-19) monoclonal antibody (1:1000; Cat. No. 3671; CST), and rabbit anti-β-actin monoclonal antibody (1:1000; Cat. No. 4970; CST). After washing, samples were incubated (1 h, room temperature) with secondary antibodies: goat anti-rabbit IgG (H&L) (1:5000; Cat. 926-32211; LI-COR) and goat anti-mouse IgG (H&L) (1:5000; Cat. 926-68070; LI-COR). After the film was scanned in the Odyssey Fluorescence Imaging System, quantitative analysis was performed by Image J Software.

### 4.14. Statistical Analysis

All results were expressed as Mean ± SEM. One-way ANOVA was used for the assessment of indicators among groups, followed by Dunnett’s test for the post hoc test. In all calculations, a difference at *p* < 0.05 was regarded as significant. Statistical analysis was performed blindly on these independent values. All data were plotted in GraphPad Prism 9 (GraphPad Software, San Diego, CA, USA).

## 5. Conclusions

In conclusion, we demonstrated that NAR was highly effective in improving pain-like behaviors, and reducing articular index scores and inflammation factors in CIA rat models. We also identified that NAR was able to inhibit the polarization state and migration capability of spleen CD4^+^ T lymphocytes, thus reducing the account of CD4^+^ T cells infiltrating the joint in RA. Taken together, this is the first time that we have clarified the contribution of the polarization and migration of CD4^+^ T cells in the pathological mechanism of RA. To our knowledge, this is also the first time that mitochondrial fission has been brought into focus in terms of its impact on the pathogenesis of RA by regulating T cell polarization and migration. More importantly, we identified that NAR might interfere with a mitochondrial diversion in CD4^+^ T cells and thus regulate their polarization and migration properties, which corroborates its potential as an alternative therapeutic option for RA.

## Data Availability

The original contributions presented in the study are included in the article/Supplementary Materials, further inquiries can be directed to the corresponding authors.

## Supplementary Materials

The following supporting information can be downloaded at: https://www.mdpi.com/article/10.3390/ijms24010279/s1.

## Abbreviations

APCs: antigen-presenting cells, CIA: Collagen induces arthritis, CXCL12: C-X-C motif chemokine ligand 12, Drp1: dynamin-related protein 1, FCA: Freund’s complete adjuvant, FIA: Freund’s incomplete adjuvant, Fis1: mitochondrial fission protein 1, ICAM1: intercellular adhesion molecule 1, IL-6: Interleukin 6, Mdivi-1: mitochondrial division inhibitor 1, MLC: myosin light chain, NAR: naringenin, RA: rheumatoid arthritis, TNF-α: Tumor necrosis factor α, TOM20: translocase of outer mitochondrial membrane 20.
