# Supplementary material for: The Flavonoid Naringenin Alleviates Collagen-Induced Arthritis through Curbing the Migration and Polarization of CD4+ T Lymphocyte Driven by Regulating Mitochondrial Fission
Source: Int J Mol Sci. 2022 Dec 23;24(1):279. doi: 10.3390/ijms24010279 (PMC9820519; doi:10.3390/ijms24010279)
Supplement: Supplementary file 1 [file ijms-24-00279-s001.zip › ijms-2057056-supplementary.pdf]

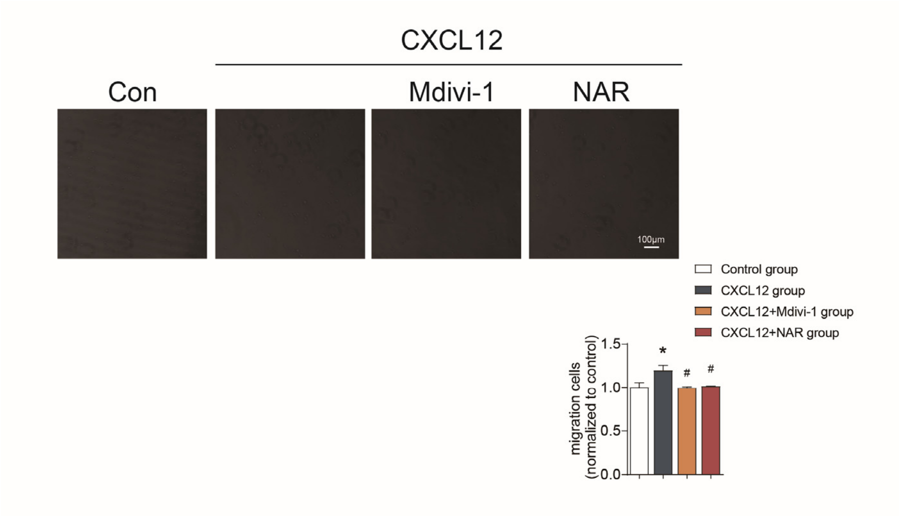

Figure S1. Transwell migration assay of different groups. The migration of CD4+ T lymphocytes in different groups was analyzed by transwell assay. * $p < 0.05$ vs. control group, # $p < 0.05$ vs. CXCL12 group, by repeated-measures one-way ANOVA followed by post hoc Dunnett's multiple comparisons test. All scale bars are 100 μm. The data are presented as means ± SEM (n = 3 mice per group).
